# More N fertilizer, more maize, and less alfalfa: maize benefits from its higher N uptake per unit root length

**DOI:** 10.3389/fpls.2024.1338521

**Published:** 2024-02-07

**Authors:** Zeqiang Shao, Congcong Zheng, Johannes Auke Postma, Qiang Gao, Jinjing Zhang

**Affiliations:** ^1^ College of Resource and Environment Engineering, Jilin Institute of Chemical Technology, Jilin, China; ^2^ College of Resources and Environmental Sciences, Jilin Agricultural University/Key Laboratory of Sustainable Utilization of Soil Resources in the Commodity Grain Bases in Jilin Province, Changchun, China; ^3^ Institute of Bio- and Geosciences – Plant Sciences (IBG-2), Forschungszentrum Jülich GmbH, Jülich, Germany; ^4^ Faculty of Agriculture, University of Bonn, Bonn, Germany

**Keywords:** maize/alfalfa intercropping, interspecific competition, N uptake, dose-response curve, root plasticity

## Abstract

Root plasticity is fundamental to soil nutrient acquisition and maximizing production. Different soil nitrogen (N) levels affect root development, aboveground dry matter accumulation, and N uptake. This phenotypic plasticity is well documented for single plants and specific monocultures but is much less understood in intercrops in which species compete for the available nutrients. Consequently, the study tested whether the plasticity of plant roots, biomass and N accumulation under different N levels in maize/alfalfa intercropping systems differs quantitatively. Maize and alfalfa were intercropped for two consecutive years in large soil-filled rhizoboxes and fertilized with 6 different levels of N fertilizer (0, 75, 150, 225, 270, and 300 kg ha^-1^). Root length, root surface area, specific root length, N uptake and yield were all increased in maize with increasing fertilizer level, whereas higher N rates were supraoptimal. Alfalfa had an optimal N rate of 75-150 kg ha^-1^, likely because the competition from maize became more severe at higher rates. Maize responded more strongly to the fertilizer treatment in the second year when the alfalfa biomass was much larger. N fertilization contributes more to maize than alfalfa growth via root plasticity responses. Our results suggest that farmers can maximize intercropping yield and economic return by optimizing N fertilizer management.

## 1 Introduction

Intercropping refers to the simultaneous growing of two or more crops in the same plot. Cereal/legume intercropping has a long history of cultivation worldwide (Chamkhi et al., 2022), Numerous studies have demonstrated the benefits of cereal/legume intercropping, including increasing crop yield and soil quality (Raseduzzaman and Jensen, 2017; Martin-Guay et al., 2018), enhancing nutrient use efficiency, reducing the need for chemical fertilizer application and plant disease incidence and pesticide application (Nawar et al., 2020; Zheng et al., 2020; Ding et al., 2021; Udayakumar et al., 2021). Northeast China is an important national production base for maize and pasture (Wang et al., 2020), accounting for approximately 15% of China’s total agricultural area (Guo et al., 2021). Crop monoculture patterns and excessive use of nitrogen fertilizers were two major challenges that seriously hamper sustainable agricultural development in the region (Thakur et al., 2023; Gao et al., 2020). Excessive application of N fertilizers has led to a number of environmental problems such as soil acidification and degradation, groundwater contamination, soil pollution and atmospheric pollution (Cruz et al., 2009; Williams et al., 2017), and also reduction of biodiversity (Hakeem et al., 2011; Cameron et al., 2013; Williams et al., 2017; Sarula et al., 2022), which in turn threaten crop production and human health through the food chain. To improve the sustainable development as well as nutrient utilization efficiency of the agriculture system, opting for cereal/legume intercropping over the extensive sole cropping system can be a promising method in this region.

Phosphorus and N utilization can be enhanced in cereal/legume intercropping through interactions between below-ground parts (Abou El-Enin et al., 2023). Root architecture, or the size and geographical distribution of the root system, is an important feature of resource acquisition in ecological and agronomic contexts (Lynch, 1995). This is because they define the total volume of soil that the plant explores and the total surface area that is exchanged between the root system and the soil solution, the size and structure of the root system have a significant role in the efficiency of nutrient uptake (Nacry et al., 2013). Recent studies on belowground root interactions in cereal/legume intercropping have been focused on efficient N utilization through root ecological niche segregation (Li et al., 2011), root contact (Brooker et al., 2015), and root secretion (Li et al., 2016). Although the single-crop dose response to N fertilizer levels has been intensively studied below- and aboveground, so far little is known about N dose-response curves in intercropping, especially in a cereal/legume system. Besides root morphological traits that contribute to the intercropping response to N fertilizer levels, biological N fixation can also make an important contribution to reducing N fertilizer inputs in intercropping systems (Ehrmann and Ritz, 2014; Rasool et al., 2021). Although most legume species can provide N for the intercropping system through biological N fixation, the intercrop still needs to be fertilized with N to maximize yield (Dilfuza et al., 2018). When the soil N content is high, N fixation by legumes can be inhibited, leading to the onset of “N block-age” (Šimon and Škrdleta, 1983). Thus, N dose-response curves in intercropping can be quite different due to cereal/legume competition, and determining the appropriate amount of N application in cereal/legume intercropping systems is important to promote sustainable agricultural development and protect the environment. In addition, the different N forms can also significantly affect root and whole-plant growth. Fertilized urea will be converted to ammonia and then to nitrate after being supplied to the soil, ammonium (NH_4_^+^-N) and nitrate (NO_3_^–^N) are the two main forms of inorganic N available to plants (Martin et al., 2010). The uptake preferences of different species tend to vary widely, with most species preferring NO_3_^–^N and only a few preferring NH_4_^+^-N (Arnon, 1937), and almost all plants grow and develop best under mixed nitrate-ammonium N supply (Schortemeyer et al., 1993). For example, Hessini et al. (2019) found that NH_4_^+^-N favored maize growth more than NO_3_^–^N. Change in the NO_3_^-^/NH_4_^+^ ratio induced by N fertilization can shape the plant response in the intercropping system.

Root architecture, however, is influenced not only by fertilization but also by neighboring plants (Hecht et al., 2019; Luo et al., 2020). Although intensive studies have recently been carried out on the dose-response curve of different crops (Paponov et al., 2005; McLachlan et al., 2020; Lecarpentier et al., 2021), there is a lack of research on the effects of different N levels on root development and N uptake and utilization in intercropping systems, particularly in maize/alfalfa intercrops. We conducted a two-year rhizoboxes experiment with continuous cropping of maize and alfalfa under different N fertilization levels and examined the root morphology, associated N uptake, biomass production, and yield. We established N fertilizer-response curves for both species and asked how their response depended on competition. This study aimed to (1) determine how N fertilizer levels influence root development and N absorption and (2) determine the link between root and shoot accumulation and N uptake in a maize/alfalfa intercropping system. This study offers a scientific foundation for attaining N fertilizer reduction and N use efficiency improvement in cereal and legume intercropping systems to achieve sustainable agricultural output.

## 2 Materials and methods

### 2.1 Study area

In 2015–2016, a greenhouse experiment was conducted with root boxes at Jilin Agricultural University (43°48′28.59″N, 125°24′50.38″E). The climate belongs to the north temperate continental monsoon category, with four distinct seasons and moderate semiarid characteristics. The growing degree days (≥10°C) are characterized by a temperature of 2 200–3 000°C. The final frost and first frost occur in April and September, respectively. The temperature, light, and air humidity conditions in the half-opened rain shelter were close to those in an open field, while the watering conditions were controlled. The average temperature and precipitation in 2015 and 2016 were 19°C and 326.8 mm and 19.8°C and 494 mm, respectively. Evaporative cooling and shade cloth were used to control the temperature on sunny days.

**Figure 1:**
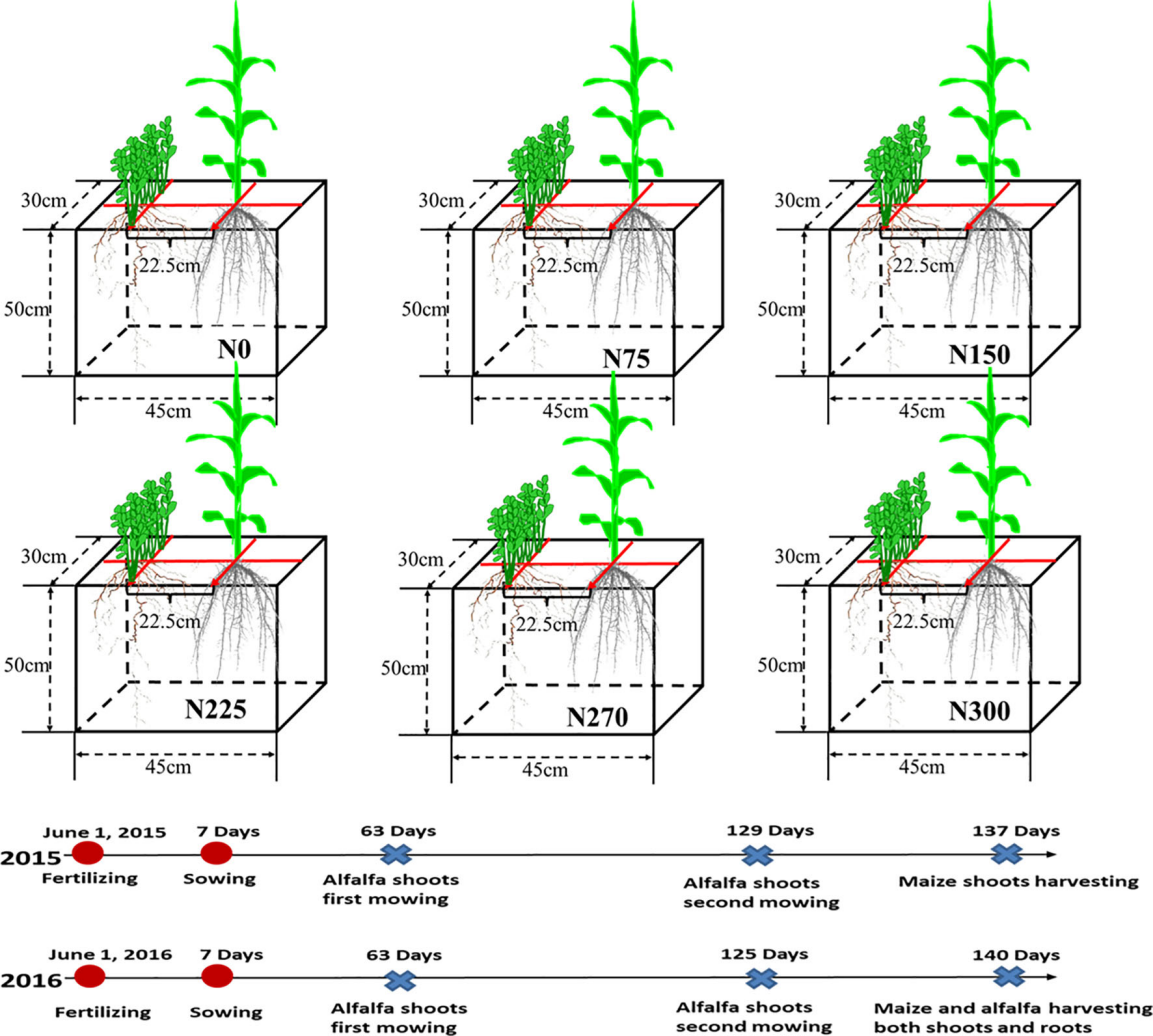
Schematic diagram of the maize/alfalfa intercropping rhizoboxes experiment. N0, N75, N150, N225, N270 and N300 represent 0 kg ha^-1^, 75 kg ha^-1^, 150 kg ha^-1^, 225 kg ha^-1^, 270 kg ha^-1^, and 300 kg ha^-1^ N fertilization treatments, respectively. The fertilizing, sowing, mowing and harvesting times in 2015 and 2016 are also marked.

Agricultural topsoil (0-20 cm) from the region was used in the experiment, and the type of soil was black soil, equivalent to typical Phaeozem in the World Reference Base (WRB) system (FAO, 2006). The soil was air-dried and sieved (3-cm aperture), and stones, root remnants, and other contaminants were removed before being used. The physical and chemical properties of soil are as follows: organic C, 21.85 g kg^-1^; total N, 1.48 g kg^-1^; alkali-hydrolysable N, 80.36 mg kg^-1^; rapidly available P, 14.82 mg kg^-1^; rapidly available K, 115.42 mg kg^-1^; and soil pH (soil: water =1:2.5), 6.46. As extractants, NaHCO_3_ (0.5 mol L^-1^) and NH_4_OAc (1 mol L^-1^) were employed to measure available P and K, respectively (Ravazzolo et al., 2021). The experiment lasted two years during which two maize crop cycles were sown while alfalfa was grown continuously (growing seasons of 2015 and 2016, **Figure 1**).

### 2.2 Experimental materials and design

The maize variety Zhengdan 958 (*Zea mays* L. cv. Zhengdan 958) and the alfalfa variety “Dongmu No. 1” (*Medicago sativa* L. cv. Dongmu No. 1) used in this study are both widely cultivated in the region. The alfalfa variety is a cold- and drought-resistant variety bred by Northeast Normal University and widely planted in the test area. This variety generally returns to green around April 15 and can be mowed for 2-3 crops per year. Zhengdan 958 is a compact maize variety with medium ears, which is a recommended variety for Jilin Province. Six levels of N application were used in the experiment: N0 (No N fertilizer of any kind), N75 (75 kg ha^-1^ N), N150 (150 kg ha^-1^ N), N225 (225 kg ha^-1^ N), N270 (270 kg ha^-1^ N), and N300 (300 kg ha^-1^ N). The control level was set at 0 kg ha^-1^ N. To fulfill crop growth requirements, identical amounts of P and K (120.0 kg ha^-1^ for P and 60.0 kg ha^-1^ for K) were fertilized into each box. Air-dried soil (51 kg; passed through a 2-mm sieve) was placed into plastic pots (length: 45 cm, width: 30 cm, height: 50 cm) (**Figure 1**). The amount of fertilizer to be applied was calculated according to the soil amount. The fertilizers used in this study were all single fertilizers, namely, urea, superphosphate, and potassium chloride, all fertilizers are mixed evenly with the soil and balanced for a week before sowing.

The experiment followed a completely randomized design with four replications of each treatment (alfalfa is a perennial leguminous forage that can be safely wintered in Northeast China). In the first year, maize was planted simultaneously on 8 June between 2015 and 2016. In the second year, maize was sown by hand next to the alfalfa, which regrew from the first-year planting (**Figure 1**). Each box was planted with two to three maize seeds at a depth of 5 cm. Shortly after emergence, the maize was thinned to one healthy seedling per pot, but thinning densities were much lower than actual production in the field experiment, as boxes were spaced far apart. The sowing volume of alfalfa was 18 kg ha^-1^ with a 2-cm seeding depth, the mass of alfalfa seed sown was 0.12 g in each pot based on the area, the alfalfa was thinned to 20 healthy seedlings per pot, and the spacing between maize and alfalfa was maintained at 22.5 cm with a 1-m distance between root boxes (**Figure 1**). The plants were watered with rainwater to maintain the soil moisture at 60–70% of the field water-holding capacity throughout the growth stage, and the soil moisture was monitored with micro tensiometers (Nanjing Canglang Technology Development Co., Ltd., China). Weeds were controlled manually using a manual weed puller, whereas pests and diseases were controlled with different insecticides and pesticides when needed during the growing season.

### 2.3 Sampling and measurements

Alfalfa was harvested during the blooming phase on 2 August and 7 October 2015 and on 2 August and 3 October 2016. Maize was harvested at maturity on 15 October 2015 and 18 October 2016 (maize roots were not collected in 2015) (**Figure 1**). All sides of the root box were cut off with a saw, large clods of soil were shaken off the root surface using the shaking method, and the root system was placed in a separate mesh bag (0.15 mm). Soil particles on the root surface were then rinsed with plenty of water, and the roots were collected. All roots were then preserved in 50% ethanol, scanned with an Epson Perfection V800 scanner (Epson, Inc., Japan) at a resolution of 23.6 pixels mm^−1^ (600 dpi), and analyzed using the image-processing software WinRHIZO Pro (Vision 5.0a, Canada, 2013) to obtain root morphology parameters including the root length, root surface area, and root volume. The fresh samples were then dried at 105°C for 30 minutes and at 75°C to a constant weight to determine the dry weight of the dried samples.

The N concentration of plants was determined by the Kjeldahl procedure after digestion with H_2_SO_4_-K_2_SO_4_-CuSO_4_ methods (Ishaq et al., 2023). NO_3_^−^ and NH_4_^+^ concentrations in rhizosphere soil were extracted with 2 M KCl and then determined by a segmented flow analysis (SFA; FUTURA, AMS, France) (Hameed et al., 2022). Soil pH was determined with a pH combination electrode in a 1:2.5 soil/distilled water suspension (Muneer et al., 2022).

N recovery efficiency was calculated as the difference in total plant content of N between a test treatment and the N0 treatment divided by the difference in the amount of N applied between the test treatment and the N0 treatment (Banik et al., 2023). N utilization efficiency, which is defined as plant biomass production per unit of N uptake (Haque et al., 2022), was calculated as total plant dry mass [shoot dry mass (SDM) plus root dry mass (RDM)] per pot divided by total plant content of N per pot in this study (Dhaka et al., 2020).


N recovery efficiency= (U-U0)/F

where U is the N uptake by the crop in the N application area, U0 is the N uptake by the crop in the non-N application area, and F is the N application (Dhaka et al., 2020).


N utilization efficiency= (SDM+RDM)/NU

where SDM is the shoot dry mass, RDM is the root dry mass, and NU is the total plant content of N per pot in this study.

The percentage of alfalfa N derived from the atmosphere (%Ndfa), based on ^15^N natural abundance method, and the equation is as follows (Wang et al., 2020):


$$\%\text{Ndfa}=100\times \frac{\delta^{15}\text{N of reference plant}-\delta^{15}{\text{N of N}}_{2}\text{ fixing plant}}{\delta^{15}\text{N of reference plant}-\text{B}}$$

Where $\delta^{15}$ N of reference plant and N_2_ fixing plant are the $\delta^{15}$ N value of maize and alfalfa, respectively, and the B-value is derived from $\delta^{15}$ N analyses of shoots of plant grown with a nutrient solution free of nitrogen; In this study, the B-value was -0.92‰ of alfalfa shoots (Wang et al., 2020).

The amount of N_2_ fixed was calculated as the product of alfalfa dry weight, %N content and %Ndfa, and the equation is as follows (Shao et al., 2021):


$${\text{Ndfa (kg N ha}}^{-1}\text{) = \%Ndfa}\times {\text{DM (t ha}}^{-1})\times {\text{Shoot N concentration (g N kg}}^{-1}\text{DM)}$$

### 2.4 Statistical analysis

The Shapiro−Wilk and Tukey’s honestly significant difference (HSD) tests (α = 0.05) were used to examine the normality and homoscedasticity of the residuals. Tukey’s HSD test (α = 0.05) was used to compare means. SAS 9.2 (SAS Institute, Inc., Cary, North Carolina, USA) was used to conduct statistical analyses using one-way analysis of variance (ANOVA). Origin Pro 2021 (Norhtampton, MA, USA) was used to evaluate correlations between belowground root morphological features, soil pH, biomass and N accumulation in the intercropping system. To detect significant changes between treatments, the international standard software CANOCO 5 and CanoDraw (Microcomputer Power, Ithaca, USA) were used for ranking procedures, and a linear model was chosen for redundancy analysis (RDA).

## 3 Results

### 3.1 Effects of N fertilization level on maize and alfalfa yields

**Figure 2:**
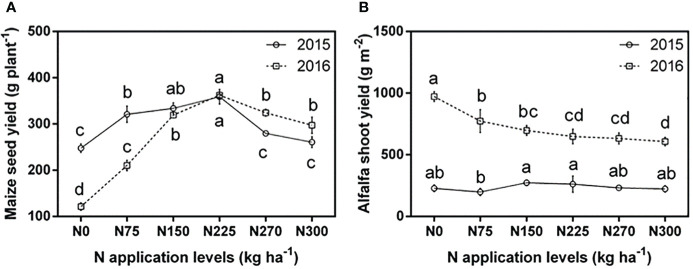
Comparisons of average maize and alfalfa yields in two consecutive years under different N application levels. **(A)** Maize yield in 2015 and 2016, **(B)** Alfalfa yield in 2015 and 2016. Different lowercase letters indicate a significant difference under six N application levels in the same year. Values = means ± SE. The data correspond to the total dry grain yield of maize and the total dry shoot biomass of alfalfa after harvest.

Maize seed yield was more responsive to N application than alfalfa biomass yield (**Figure 2**). In both years, maize yield was maximal at N225 and significantly declined at N270 (2015: 247.37-333.72 g plant^-1^; 2016: 121.22-362.29 g plant^-1^). This decline was not because of competition, as alfalfa had a maximal yield at lower N levels (N150 and N75 in 2015, and 2016 respectively) (2015: 197.61-273.28 g m^2^; 2016: 607.35-971.39 g m^2^). Both species had a stronger response to N application in the second year, in which the total biomass per box was much greater, and presumably the competition was much stronger.

### 3.2 Effects of N fertilization level on maize and alfalfa N accumulation

**Figure 3:**
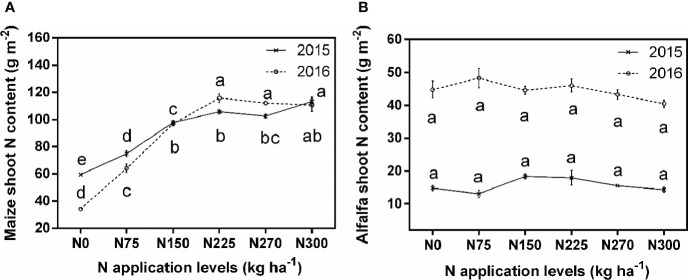
Comparisons of average maize and alfalfa N content in the shoot in two consecutive years under different N application levels. **(A)** Maize N content in the shoot in 2015 and 2016, **(B)** Alfalfa N content in the shoot in 2015 and 2016. Different lowercase letters indicate a significant difference under six N application levels in the same year. Values = means ± SE. The data correspond to maize and alfalfa after harvest.

The fertilizer dose-response curve for shoot N, which is presumably representative of the total N acquisition, closely followed the yield response curve. At high N levels, maize acquired more N than alfalfa, but its N acquisition declined much more at sub- and supraoptimal levels, and similar to the yield, this decline was even stronger in the second year when alfalfa acquired more N (**Figures 3A, B**). In 2016, compared to N0, maize contained 225% more N in the shoot when optimally fertilized, whereas for alfalfa the difference was only 45%.

### 3.3 Effects of N fertilization level on root plasticity of maize and alfalfa

**Figure 4:**
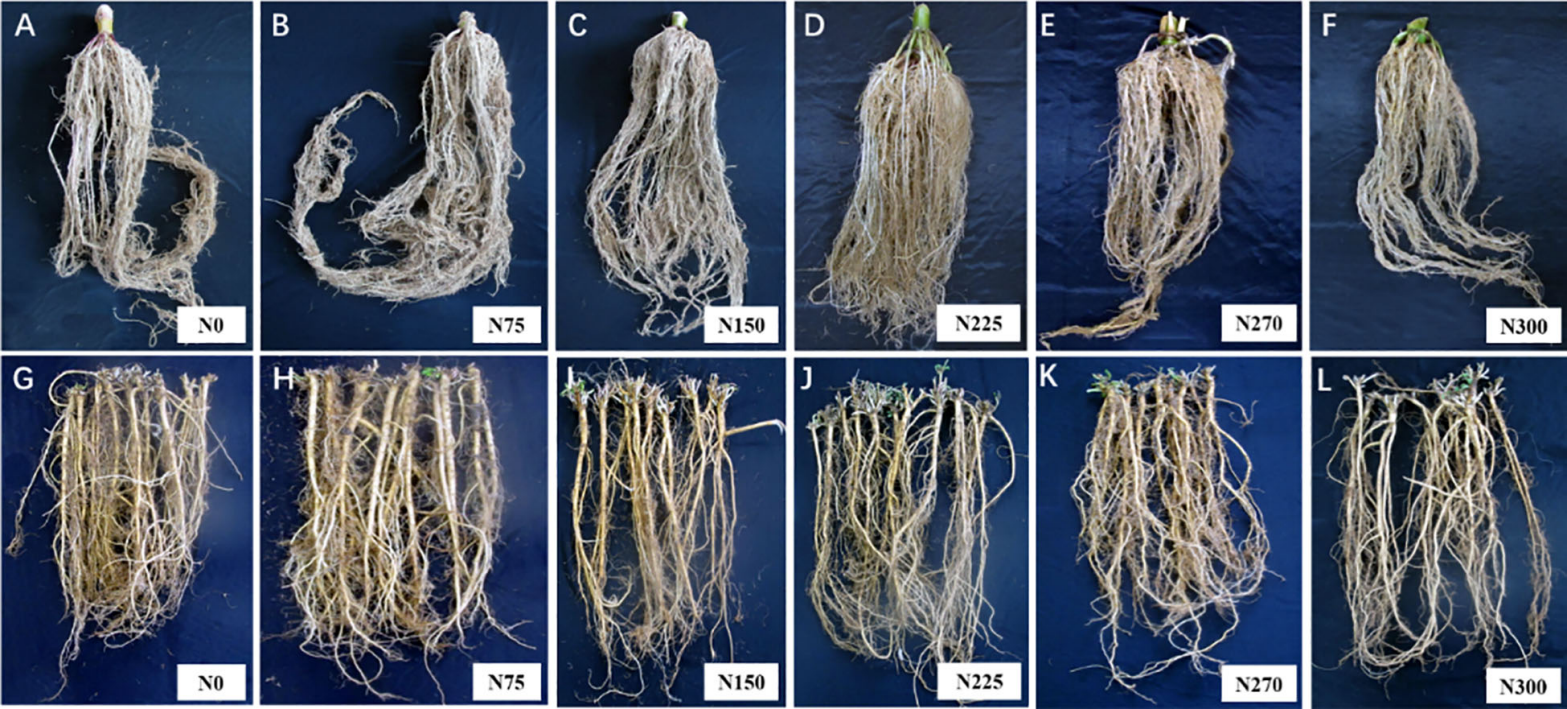
Root morphology of maize and alfalfa grown with different N application levels. **(A–F)**, Maize root morphology under 0 kg ha^-1^, 75 kg ha^-1^, 150 kg ha^-1^, 225 kg ha^-1^, 270 kg ha^-1^, and 300 kg ha^-1^ N fertilization treatments, respectively. **(G–L)**, alfalfa root morphology under 0 kg ha^-1^, 75 kg ha^-1^, 150 kg ha^-1^, 225 kg ha^-1^, 270 kg ha^-1^, and 300 kg ha^-1^ N fertilization treatments, respectively. The data correspond to the second year after the maize and alfalfa harvest.

**Figure 5:**
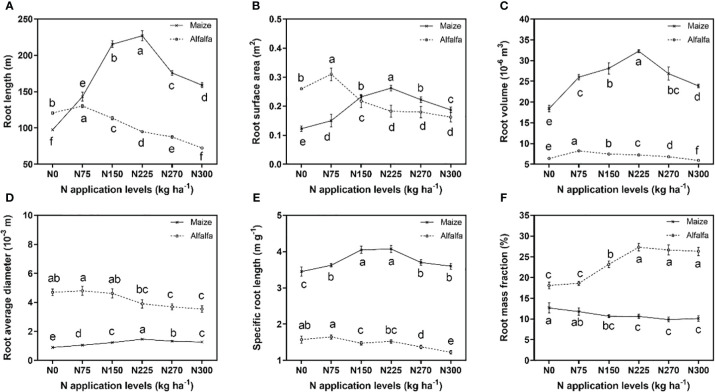
Comparisons of root length **(A)**, root surface area **(B)**, root volume **(C)**, root average diameter **(D)**, specific root length **(E)**, and root mass fraction **(F)** of maize and alfalfa in 2016 under different N application levels. Different lowercase letters indicate a significant difference under six N application levels in the same year. Values = means ± SE. The data correspond to maize and alfalfa after harvest.

The application of N had distinct impacts on the root morphology of maize and alfalfa (**Figures 4**, **5**). All indicators of root morphology for both maize and alfalfa showed an increasing and then decreasing trend with increasing N application in 2016 (**Figure 5**). For maize, compared to the N0 treatment, root length significantly increased by 46.74%, 120.64%, 132.56%, 80.07% and 62.86% under the N75, N150, N225, N270, and N300 treatments, respectively. The root surface area increased by 23.23%, 92.11%, 115.17%, 80.55%, and 56.04%, respectively. Root volume increased by 41.08%, 52.65%, 75.07%, 45.71%, and 29.82%, respectively. The average root diameter increased by 17.55%, 37.33%, 62.95%, 47.91%, and 40.95%, respectively. The specific root length increased by 5.07%, 17.39%, 18.12%, 7.25%, and 4.35%, respectively. In addition, under the N225 treatment, all the parameters reached their maximum levels.

In alfalfa, the root length, root surface area, root mean diameter, root length density, and specific root length increased by 7.96%, 17.59%, 2.18%, 8.33%, and 4.76% under the N75 treatment compared to the N0 treatment, respectively. There was a significant decrease in root length (but not root volume) under the N150, N225, N270, and N300 treatments compared to the N0 treatment, and the root length was reduced by 6.02%, 21.48%, 27.36%, and 40.06%, respectively. The root surface area was reduced by 17.30%, 30.57%, 31.26%, and 38.63%, respectively. The average root diameter was reduced by 1.49%, 16.73%, 21.31%, and 24.45%, respectively. The specific root length was reduced by 6.35%, 3.17%, 12.70%, and 22.22%, respectively. Root volume increased by 6.94%, 22.22%, 27.78%, and 40.28% under the N75, N150, N225, and N270 treatments, respectively, and decreased only under the N300 treatment, by 7.06% compared to the N0 treatment.

### 3.4 Effects of N fertilization level on maize and alfalfa N recovery and utilization efficiency

**Figure 6:**
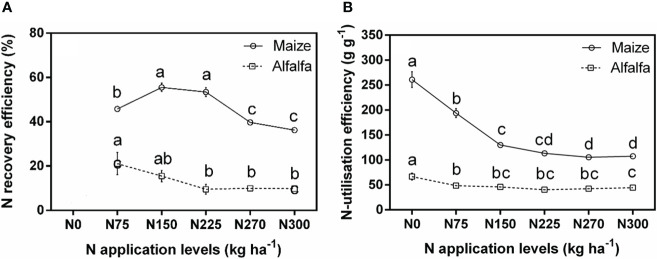
Comparisons of maize and alfalfa N recovery efficiency **(A)** and N utilization efficiency **(B)** in 2016 under different N application levels. Different lowercase letters indicate a significant difference under six N application levels in 2016. Values = means ± SE.

Different N application rates also had a significant effect on the N recovery efficiency (**Figure 6**). For maize, the N recovery efficiency showed an increasing and then decreasing trend with increasing N application. The N recovery efficiency was maximum under the N225 treatment among all six N application levels (2016: 53.4%). For alfalfa, N recovery efficiency was maximum under the N75 treatment (2016: 21.11%).

Different N application rates also had a significant effect on the N utilization efficiency. The N utilization efficiency of maize and alfalfa showed a decreasing trend with increasing N application. For maize, N utilization efficiency decreased by 25.74%, 50.26%, 56.25%, 59.65%, and 58.82% compared to the N0 treatment under the six N application levels, respectively. For alfalfa, N utilization efficiency decreased by 2.16%, 24.01%, 41.28%, 32.85%, and 30.28% compared to the N0 treatment under the six N application levels, respectively.

### 3.5 Effects of N fertilization level on alfalfa N fixation rate and amount of N fixation

%Ndfa and Ndfa of alfalfa were significantly affected by N level. The %Ndfa under different N levels was significantly reduced by 6.96% and 37.39% compared to N0 treatment. However, the Ndfa was slightly increased by 1.43% and 1.95% under N75, N150 and N225 treatments compared to N0 treatment, and was significantly reduced by 21.69% and 37.69% under N270 and N300 treatments (**Table 1**).

**Table 1:** Effects of N application levels on the percentage of N derived from atmosphere (%Ndfa) and amount of N_2_ fixed (Ndfa) of alfalfa at the second flowering stage of alfalfa.

N level	%Ndfa	Ndfa (Kg N ha^-1^)
N0	44.77 ± 0.39a	58.54 ± 3.61a
N75	41.65 ± 4.20a	59.38 ± 724a
N150	35.33 ± 2.60b	59.56 ± 4.73a
N225	33.67 ± 3.50b	59.68 ± 4.81a
N270	30.85 ± 5.56bc	45.84 ± 7.47b
N300	28.03 ± 1.86c	36.48 ± 3.52c

Different lowercase letters indicate significant differences between six N levels in 2015.

### 3.6 Relationships between maize and alfalfa root morphological indices and N uptake

**Figure 7:**
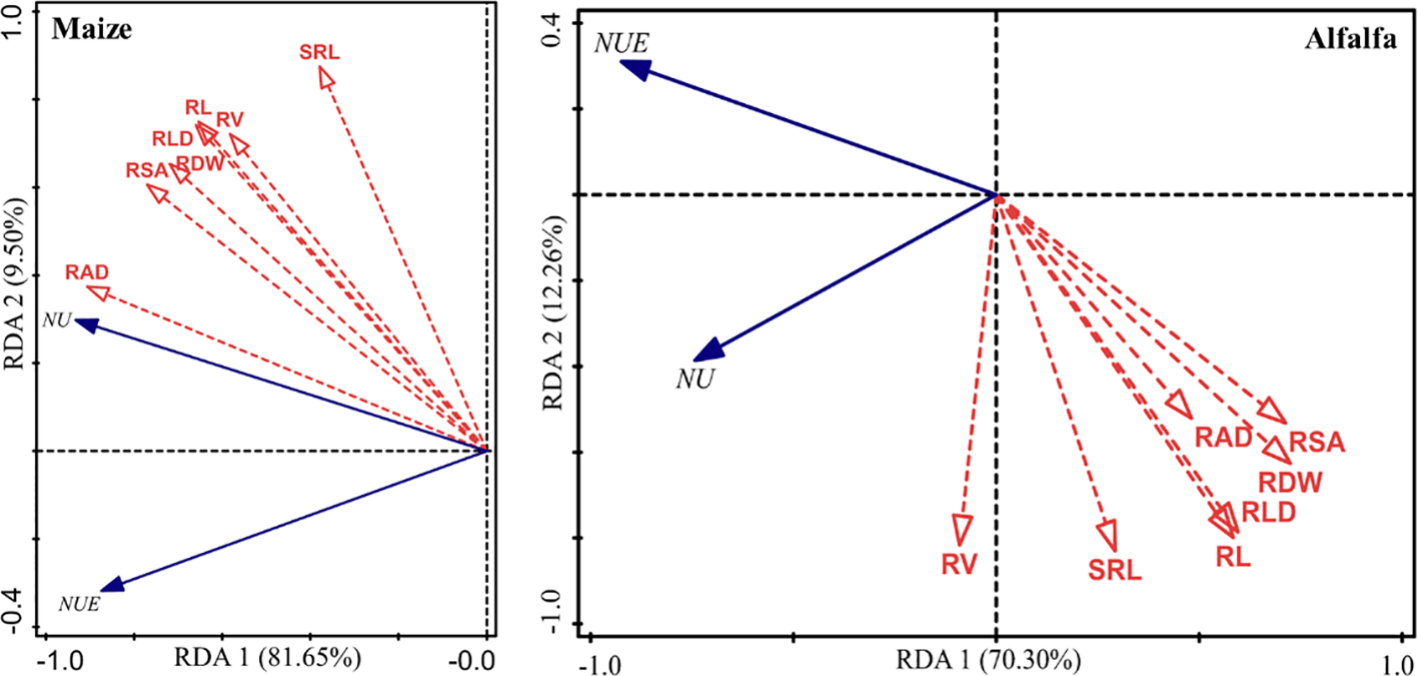
Redundancy analysis (RDA) of the relationships between N uptake and the root length (RL), root surface area (RSA), root volume (RV), root average diameter (RAD), root length density (RLD), specific root length (SRL) and root dry weight (RDW). NU and NUE represent the N uptake and N uptake efficiency, respectively, in the maize/alfalfa intercropping system in 2016.

Redundancy analysis (RDA)showed that the root length (RL), root surface area (RSA), root volume (RV), root average diameter (RAD), root length density (RLD), and specific root length (SRL) of maize roots could explain 81.65% and 9.50% of the variation in the RDA1 and RDA2 axes, respectively (**Figure 7**). Among all the bound variables, the RAD, SRL, and root dry weight (RDW) of maize had a significant association with N uptake (P=0.002, 0.002, and 0.044), explaining 68.4%, 17.2% and 2.3% of the variance, respectively (**Table 2**).

**Table 2:** Results of the permutation test of the redundancy analysis (RDA) on predictored variables of N uptake in maize and alfalfa.

Maize				Alfalfa			
Variable	Explains (%)	F value	P value	Variable	Explains (%)	F value	P value
RAD	68.4	47.7	0.002	RDW	41.7	15.7	0.002
SRL	17.2	25.3	0.002	RV	31	23.9	0.002
RV	1.7	2.8	0.128	RSA	4.6	4.1	0.046
RDW	2.3	4.4	0.044	RLD	4.4	4.6	0.036
RSA	0.7	1.4	0.246	SRL	0.6	0.6	0.492
RL	0.6	1.2	0.264	RAD	0.2	0.2	0.782
RLD	<0.1	<0.1	0.944	RL	<0.1	<0.1	0.906

RL, RSA, RV, RAD, SRL, RDW, and RLD represent the root length, root surface area, root volume, root average diameter, specific root length, root dry weight, and root length density, respectively.

The RDA also showed that the root length (RL), root surface area (RSA), root volume (RV), root average diameter (RAD), root length density (RLD), specific root length (SRL), and root dry weight (RDW) of the alfalfa root system explained 70.30% and 12.26% of the variation in RDA1 and RDA2, respectively (**Figure 7**). Among all variables, the RDW, RV, RSA, and RLD of alfalfa had significant effects on N uptake (P=0.002, 0.002, 0.046, and 0.034), explaining 41.7%, 31.0%, 4.6% and 4.4% of the variance, respectively (**Table 2**).

## 4 Discussion

### 4.1 Fertilization increases maize yield but decreases alfalfa yield

Positive effects of cereal/legume polyculture and fertilization on plant growth, yield and nutrient uptake have been well-documented in previous studies, mainly attributing to root (root morphology and distribution) (Sun et al., 2019) and rhizosphere (root exudation and legume N fixation) (Shao et al., 2020; Wang et al., 2020) changes, as well as interspecific complementarity in space and time (Brooker et al., 2015; Li et al., 2021). As expected, N application increased maize yield by 5.32%-45.12%, but alfalfa yield peaked at low N rates of N150 and decreased significantly at higher N rates of N270 and N300 (-1.72% to -2.22%) (**Figure 2**). The possible reason is that excessive N fertilizer application inhibits the growth of rhizobia (Shao et al., 2020). In the second year, the increase in N application increased maize yield by 73.40%-198.87% in 2016, and alfalfa yield was reduced by 20.46%-37.48% in the second year compared to the N0 treatment. Interspecific competition and N application in the maize/alfalfa intercropping system promoted the growth of maize but inhibited the growth of alfalfa. Our results are in agreement with those experiments in the maize/soybean intercropping system, where N application can suppress soybean growth at certain densities (Batista et al., 2021). Maximizing maize and alfalfa yield can be accomplished by optimizing N fertilizer levels.

As alfalfa is a perennial legume and usually grows larger in the second year than in the first year, the steeper dose response of maize yield in 2016 than in 2015 indicated a greater sensitivity to N fertilization in the second year, mainly explained by the increased interspecific competition, proper N fertilizer improves both. The highest maize and alfalfa yields were reached not at the highest N fertilizer level, similarly, Zhang et al. (2017) studied soybean/maize intercropping systems in the karst region of Southwest China and showed that the total biomass and yield of the intercropping system also reached a maximum yield, but at an N application level of 450 kg ha^-1^. However, a significant decrease in biomass and yield of the intercropping system occurred at an N application of 600 kg ha^-1^. The optimal N application was much higher than that in our experiment, probably due to the low soil fertility in the karst region of Southwest China. N demand for plant growth can differ among species in both sole and polyculture systems. In our experiment, the grain yield of maize increased and then decreased with increasing N application, reaching a maximum in the N225 treatment in both years (2015: 333.72 g plant^-1^; 2016: 362.29 g plant^-1^) (**Figure 2**). For alfalfa yield, their response to N application was less pronounced and differed in two years, with a yield maximum in the N150 treatment (273.28 g m^-2^) in 2015 and a yield maximum in the N0 treatment in 2016 (971.39 g m^-2^) (**Figure 2**). This is because in 2015, alfalfa was a smaller plant with presumably lower N content, whereas it was relative larger and more competitive in 2016, especially at lower N fertilization levels. The sensitivity of maize and alfalfa to N fertilizer increased with the years. It can be predicted that the more pronounced response to N will appear in the third and fourth year under field conditions, with a larger and more competitive individual plant of alfalfa.

### 4.2 Fertilizer application increases N uptake in maize but reduces N uptake, %Ndfa and Ndfa in alfalfa

Similar to the yield response patterns, N uptake also increases and then decreases with N fertilizer levels. Compared with the N0 treatment, increased N levels increased the N uptake of maize and alfalfa, a maximum N uptake of maize can be obtained at the N225 treatment (**Figure 3**), this result is consistent with the findings of a three-year maize/soybean field experiments by Du et al. (2022), who also found that an appropriate increase in N fertilizer application could significantly affect crop N uptake. Proper N fertilization increases the chlorophyll content, enzyme activity, and photosynthesis of plant leaves, thereby promoting crop yield and N uptake (Shah et al., 2021). However, too much N fertilizer reduced the yield, biomass, and uptake of N by the crop (**Figures 2**, **3**). Possible reasons are excessive N in maize leaves decreases stomatal conductance and regulate stomata closure, thus negatively influencing photosynthetic parameters (Ahmad et al., 2022). To maximum N uptake, the optimal N application rate should be 225 kg ha^-1^ for maize and between 75 and 150 kg ha^-1^ for alfalfa (N uptake by alfalfa was maximized by an optimal N application rate of 150 kg ha^-1^ in 2015 and 75 kg ha^-1^ in 2016).

Biological nitrogen fixation is an important source of N for leguminous crops, fixed N by legume is a guarantee for achieving industrial N fertilizer reduction and sustainable agricultural development. Many studies have shown that N fixation rates in legume crops range from 0% to 90% with a mean value of 36% (Wang et al., 2020). In our study, the %Ndfa in alfalfa was in the range of 28.03% to 44.77% with a mean value of 35.72% (**Table 1**). Meantime, a significant decrease in %Ndfa of alfalfa occurred with increased N application. This can be explained by lower interspecific competition of N and decreased N fixation ability with higher N fertilizer levels. Similar results were also found in maize/alfalfa field experiments reported by Wang et al. (2020). To reduce nitrogen fertilizer waste, maximum Ndfa, and crop yield, a certain amount of N fertilizer needs to be applied in different years considering the variation of N fixation ability by alfalfa.

### 4.3 Maize benefits from its higher N uptake per unit root length than alfalfa

The root system is the main organ for synthesizing and transporting physiological activators (Nasar et al., 2022), and the plasticity of plant roots is closely related to the uptake and utilization of N (Lynch, 1995). It was found that moderate N application promoted root growth, while excessive N fertilizer inhibited root growth (Forde and Lorenzo, 2001). In agreement with Chen et al. (2020), our results showed that the level of N application can significantly influence root growth, and N effects can be quite different between different root traits of maize and alfalfa. Compared to the N0 treatment, N fertilization significantly increased root length by 46.74% to 120.64%, root surface area by 23.23% to 115.17%, root volume by 29.82% to 75.07%, root density by 50.88% to 136.84% of maize (**Figures 4**, **5**). However, for the legume species alfalfa, only the N75 treatment increased the root morphological traits of alfalfa among the six N application levels, ranging from 2.18% to 17.59%, while the N150-N300 treatments reduced root exploration, with decreases in root length of 6.02% to 40.06%, root surface area of 17.30% to 38.63%, root length density of 6.94% to 40.28% (**Figure 5**), indicating a higher root plasticity of maize than alfalfa in the maize/alfalfa intercropping system. As maize is the dominant species in cereal/legume polyculture and more rely on N fertilization (Li et al., 2006), it can benefit from its higher N uptake per unit root length than alfalfa, exhibiting a similar N accumulation with alfalfa under N0 but was 7 times higher than alfalfa when supplied with enough N. Besides N fertilization level, changes in the NO_3_^-^/NH_4_^+^ ratio and soil pH are also well-known as important factors for N uptake and root response. Alfalfa and maize’s abilities also differ in nitrate and ammonium uptake, nitrification inhibition, and N fixation. Although the soil NO_3_^-^/NH_4_^+^ ratio is dynamic and is difficult to quantify, we found a reduced NO_3_^-^/NH_4_^+^ ratio with increasing fertilization at the end of the experiment (**Supplementary Figure A1**), which may be related to the fact that maize is a species that inhibits nitrification (Otaka et al., 2022). Concurrently, the pH of the soil was highest at N225 (pH=6.8). Soil acidification and the lower NO_3_^-^/NH_4_^+^ ratio may inhibit root elongation and expansion and then reduce intercropping yield, which needs further testing to fill knowledge gaps regarding these aspects.

### 4.4 Nitrogen fertilization contributes more to maize than alfalfa growth via root plasticity responses

Maize and alfalfa biomass and N acquisition respond differently to the soil N concentrations, which is usually associated with the intercrops root plasticity response N fixation, nodulation, and N facilitation provided by alfalfa (Shao et al., 2020; Nasar et al., 2022). A field cotton experiment carried out by Chen et al. (2020) showed that increased shoot and boll biomasses were correlated with a significant improvement in the root system under the moderate N treatment. In our study, the root morphological traits were positively correlated with the aboveground biomass and yield of both maize and alfalfa (**Supplementary Figure A2**), further RDA results showed that the RAD, SRL, and RDW of maize were significantly associated with N uptake and utilization, the RDW, RV, RSA, and RLD of alfalfa were positively correlated with on N uptake (**Figure 7**). To promote efficient N uptake and utilization by plants, genetic modification on these root morphology traits can be promising in exploring plant potential and efficient nutrient utilization.

## 5 Conclusions

N fertilization enhances maize yield but reduces long-term alfalfa yield in intercrops. The responses of maize and alfalfa to different N application levels are different and should be treated separately in actual production, with the optimum fertilizer application rate being 225 kg ha^-1^ for maize and 75-150 kg ha^-1^ for alfalfa. The sensitivity of maize and alfalfa to N was increased in the intercropping system due to the increased competition in maize and alfalfa intercropping, with maize taking up and using the applied N fertilizer and the N fixed by alfalfa and alfalfa using more of its own fixed N. Belowground root parameters were significantly correlated with aboveground biomass, N uptake, and N utilization, and the higher yield of maize than alfalfa resulted from the higher N uptake per unit root length of maize. Our results suggest that regulating the root plasticity of maize and alfalfa via N application is important for theoretically improving the mechanism of efficient N utilization in cereal/legume intercropping and practically achieving N reduction and improved N use efficiency in agricultural production to ensure food security.

## Data availability statement

The raw data supporting the conclusions of this article will be made available by the authors, without undue reservation.

## Author contributions

ZS: Formal analysis, Data curation, Writing – original draft. CZ: Investigation, Writing – original draft, Writing – review & editing. JP: Supervision, Writing – review & editing. QG: Conceptualization, Funding acquisition, Writing - review and editing for this author. JZ: Supervision, Writing – review & editing.
