# Supplementary material for: More N fertilizer, more maize, and less alfalfa: maize benefits from its higher N uptake per unit root length
Source: Front Plant Sci. 2024 Feb 7;15:1338521. doi: 10.3389/fpls.2024.1338521 (PMC10879570; doi:10.3389/fpls.2024.1338521)
Supplement: Supplementary file 1 [file Image_1.pdf]

Appendix

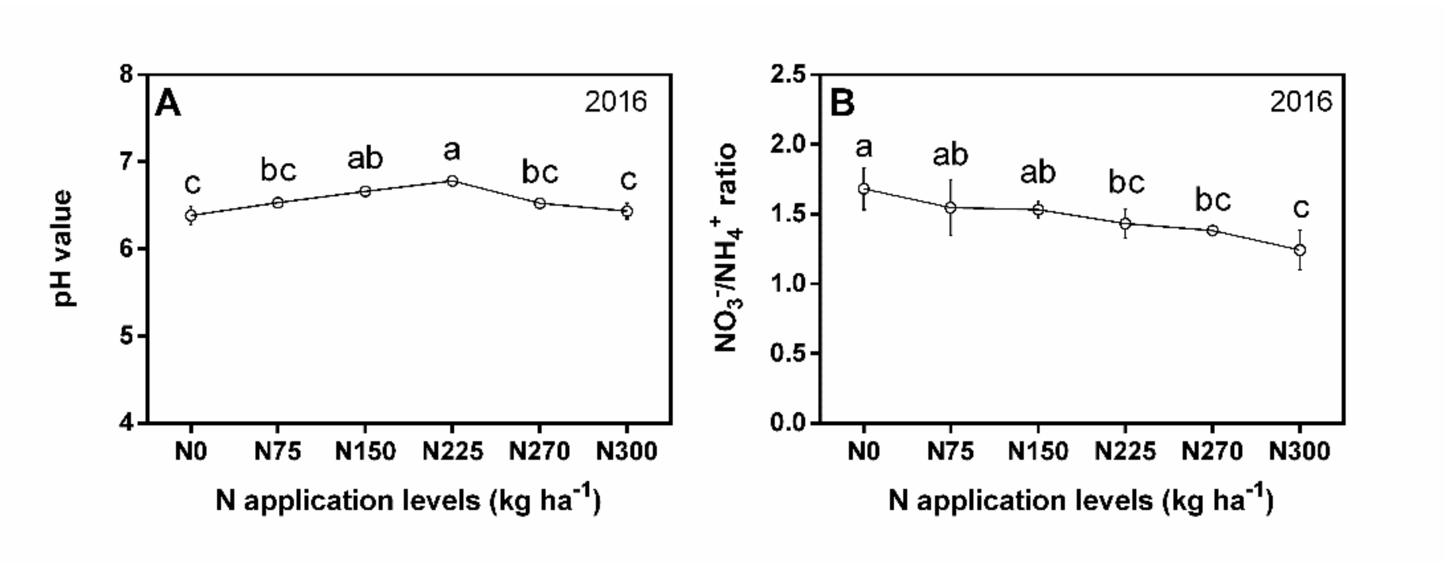

Figure A1. Comparisons of average soil pH value and NO_3^-/NH_4^+ ratio in the shoot in two consecutive years under different N application levels. A = soil pH value in 2016. B = soil NO_3^-/NH_4^+ ratio in 2016. Different lowercase letters indicate a significant difference under 6 N application levels in the same year. Values = means ± SE. These data are for maize and alfalfa after harvest.

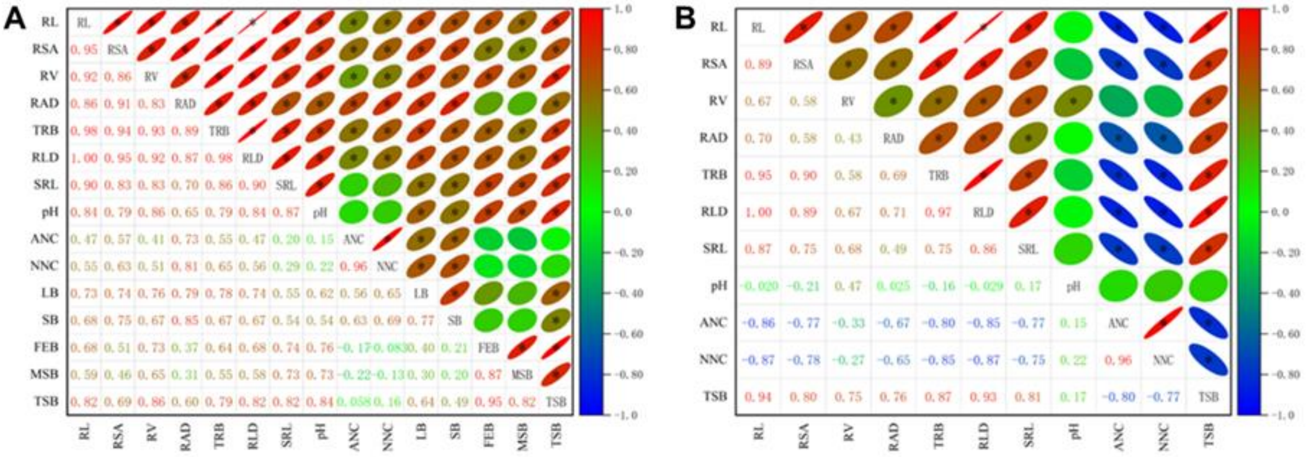

Figure A2. Corelation coefficients among the root morphology indexes and shoot biomass (A: Maize in 2016; B: Aalfalfa in 2016). RL, root length; RSA, root surface area; RV, root volume; RAD, root average diameter; RLD, root length density; SRL, specific root length; ANC, ammonium nitrogen content; NNC, nitrate N concentration; LB, leaf biomass; SB, stem biomass; FEB, female ears biomass; MSB, maize seed biomass; TSB, total shoot biomass. *Significant at $P \leq 0.05$.
